# Supplementary material for: Enzyme-controlled, nutritive hydrogel for mesenchymal stromal cell survival and paracrine functions
Source: Commun Biol. 2023 Dec 14;6:1266. doi: 10.1038/s42003-023-05643-y (PMC10719273; doi:10.1038/s42003-023-05643-y)
Supplement: Supplementary file 2 — Supplementary Information [file 42003_2023_5643_MOESM2_ESM.pdf]

Supplementary Figures and Tables

Supplementary Fig. 1: The characterization of hMSCs after near-anoxia treatment

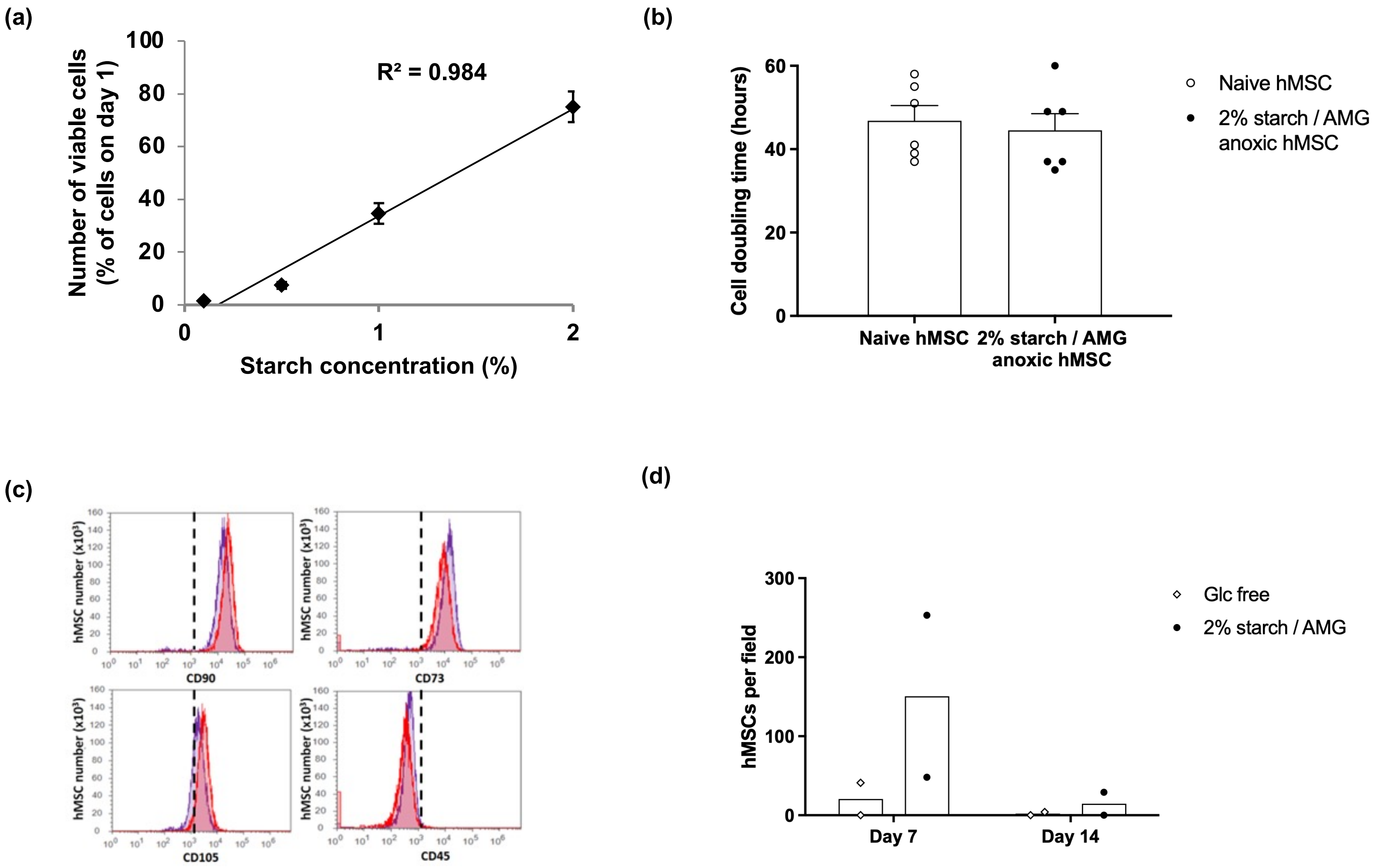

(a). Linear proportionality relation (R^2= 0.984) between the number of viable hMSCs maintained in the starch/AMG hydrogels under near anoxia conditions for 7 days of culture as a function of the initial starch concentrations (n=9). (b). Population doubling time of hMSCs cultured into the starch 2%/AMG hydrogels, in near anoxia for 14 days (i.e., Starch/AMG anoxic hMSCs), digested out from the hydrogels and transferred back to standard 2D-cell culture conditions for 10 days, and compared to the one of hMSCs maintained under standard cell culture conditions (i.e., naive hMSCs) (n=6). (c). Flow cytometry characterization of hMSCs maintained in the starch 2%/AMG hydrogels under near-anoxia conditions (purple histogram) or of naïve hMSCs cultured under standard conditions (red histogram) for 14 days (n=6). **(d)** Quantification of viable hMSCs seeded in either glucose-free hydrogels or starch 2%/AMG hydrogels after 7 and 14 days of subcutaneous implantation in nude mice after immunostaining with human β2-microglobulin (a membrane protein that stains human cells)[28] (n=2).

Supplementary Fig. 2: Starch 2%/AMG hydrogels are more effective than glucose-free hydrogels in extending the *in vitro* survival of ADSCs and myoblasts in near anoxia

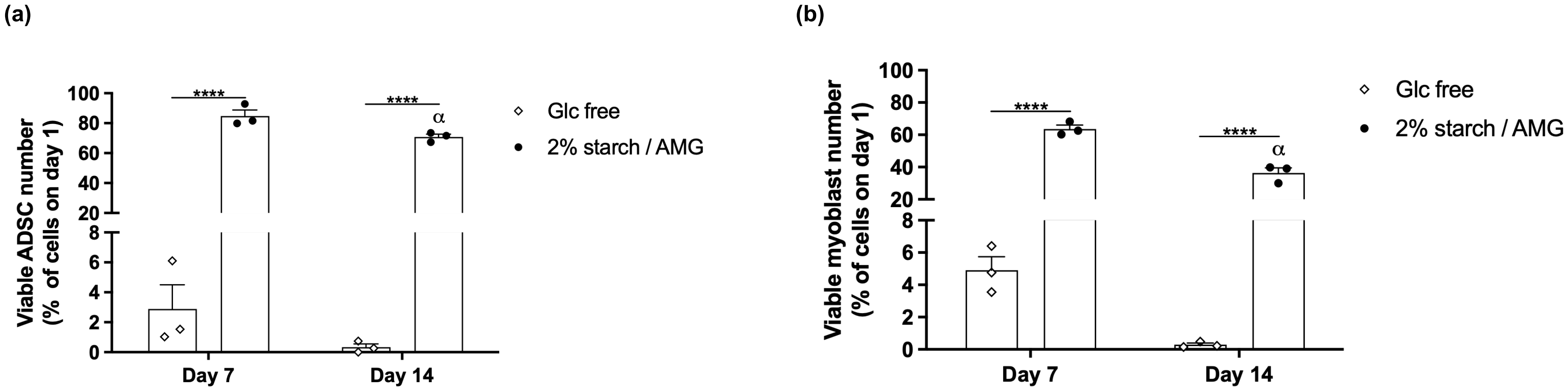

(a). Viability of human ADSCs seeded in either glucose-free or starch 2%/AMG hydrogels and exposed to near-anoxia for 7 and 14 days (n=3). (b). Viability of human myoblasts seeded in either glucose-free hydrogels, or starch 2%/AMG hydrogels, and exposed to near anoxia for 7 and 14 days (n=3).

Supplementary Fig. 3: Starch/AMG hydrogels exhibit a residual starch on day 14 post-implantation

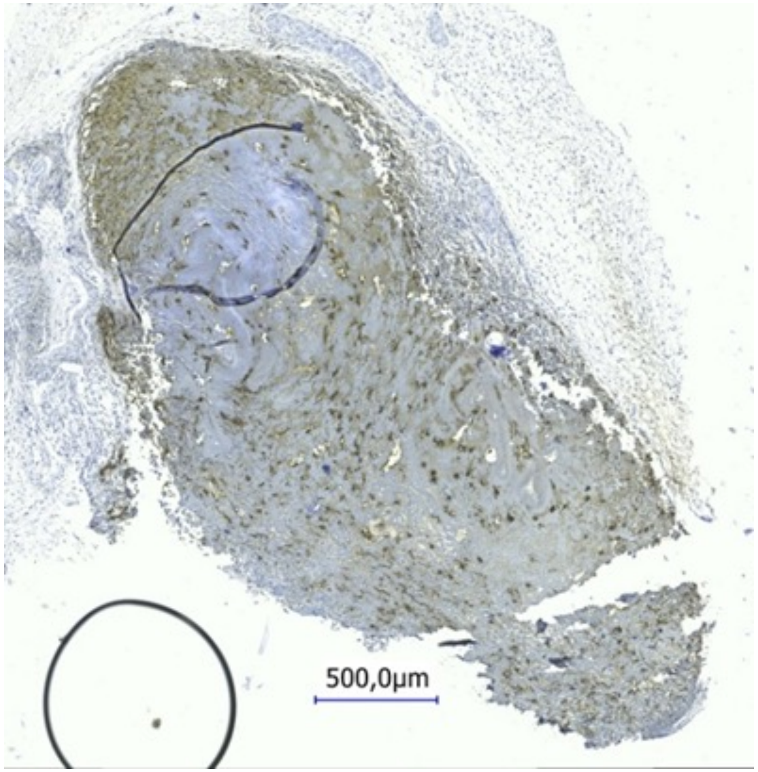

A representative image of histological section of starch/AMG hydrogel showing a residual starch that appears purplish after lugols iodine staining. Scale bar: 500 µm.

Supplementary Table 1: Composition of the hydrogels used in the present study.

		100 µl Hydrogels [a]				
**Composition**	Mix	Glc free	Glc 5.5 mM	Starch	Starch/AMG	Final Concentration
**Fibrinogen (50 mg/mL)**	1	36 µL	36 µL	36 µL	36 µL	18 mg/mL
**HEPES (10 mM)**	1	5 µL	5 µL	5 µL	5 µL	0.5 mM
**Aprotinin (3000 U/mL)**	1	3.7 µL	3.7 µL	3.7 µL	3.7 µL	112 U/mL
**hMSCs number**	1	10^5 [b]	10^5 [b]	10^5 [b]	10^5 [b]	10^6 cells/mL [b]
**Thrombin (500 U/mL)**	2	3.6 µL	3.6 µL	3.6 µL	3.6 µL	18.6 U/mL
**HEPES (10 mM)**	2	46.7 µL	46.4 µL	1.7 µL	NA	5.1 mM
**NaCl (3 M) $CaCl_2$ (0.4 M)**	2	5 µL	5 µL	NA	NA	NaCl (0.15 M) $CaCl_2$ (0.02 M)
**Glc (2,750 mM)**	2	NA	0.2 µL	NA	NA	5.5 mM
**Wheat Starch (0.2, 1, 2 or 4% (w/v))**	2	NA	NA	50 µL	50 µL	0.1, 0.5, 1 or 2% (w/v)
**AMG (117 mg/mL)**	2	NA	NA	NA	1.7 µL	136 U/mL

The indicated volumes correspond to a 100 µL hydrogel sample used for the *in vitro* experiments. [a] For 125 µl hydrogels and 200 µl hydrogels, each volume was increased 1.25- and 2-fold, respectively. [b] 1.25×10^6 hMSCs were loaded into 125 µL hydrogels for the *in vivo* assessment of the proangiogenic potential of the hMSC secretome. In contrast, 2×10^5 Luc-ZS Green-hMSCs were loaded in 200µl hydrogels for the *in vivo* assessment of hMSC survival. Glc = Glucose; NA = Not Applicable.
