# Supplementary material for: Enzyme-controlled, nutritive hydrogel for mesenchymal stromal cell survival and paracrine functions
Source: Commun Biol. 2023 Dec 14;6:1266. doi: 10.1038/s42003-023-05643-y (PMC10719273; doi:10.1038/s42003-023-05643-y)
Supplement: Supplementary file 3 — Description of Additional Supplementary Files [file 42003_2023_5643_MOESM3_ESM.pdf]

Description of Additional Supplementary Files

File name: Supplementary Data

Description: Source data behind the graphs in Figures 1-5 and Supplementary Figures 1 and 2.
