# Supplementary material for: Enzyme-controlled, nutritive hydrogel for mesenchymal stromal cell survival and paracrine functions
Source: Commun Biol. 2023 Dec 14;6:1266. doi: 10.1038/s42003-023-05643-y (PMC10719273; doi:10.1038/s42003-023-05643-y)
Supplement: Supplementary file 5 — Reporting Summary [file 42003_2023_5643_MOESM5_ESM.pdf]

nature portfolio

Corresponding author(s): COMMSBIO-23-1039-A

Last updated by author(s): Aug 23, 2023

Reporting Summary

Nature Portfolio wishes to improve the reproducibility of the work that we publish. This form provides structure for consistency and transparency in reporting. For further information on Nature Portfolio policies, see our Editorial Policies and the Editorial Policy Checklist.

Statistics

For all statistical analyses, confirm that the following items are present in the figure legend, table legend, main text, or Methods section.

n/a	Confirmed	
☒	☐	The exact sample size (*n*) for each experimental group/condition, given as a discrete number and unit of measurement
☐	☒	A statement on whether measurements were taken from distinct samples or whether the same sample was measured repeatedly
☐	☒	The statistical test(s) used AND whether they are one- or two-sided<br>*Only common tests should be described solely by name; describe more complex techniques in the Methods section.*
☐	☒	A description of all covariates tested
☒	☐	A description of any assumptions or corrections, such as tests of normality and adjustment for multiple comparisons
☐	☒	A full description of the statistical parameters including central tendency (e.g. means) or other basic estimates (e.g. regression coefficient) AND variation (e.g. standard deviation) or associated estimates of uncertainty (e.g. confidence intervals)
☐	☒	For null hypothesis testing, the test statistic (e.g. *F*, *t*, *r*) with confidence intervals, effect sizes, degrees of freedom and *P* value noted<br>*Give P values as exact values whenever suitable.*
☒	☐	For Bayesian analysis, information on the choice of priors and Markov chain Monte Carlo settings
☒	☐	For hierarchical and complex designs, identification of the appropriate level for tests and full reporting of outcomes
☒	☐	Estimates of effect sizes (e.g. Cohen's *d*, Pearson's *r*), indicating how they were calculated

Our web collection on statistics for biologists contains articles on many of the points above.

Software and code

Policy information about availability of computer code

Data collection	The bioactive factors levels were acquired using the MasterPlex QT 1.0 system (MiraiBio, Alameda, USA). The definition of viable and dead cells were acquired using Attune flow cytometer.
Data analysis	GraphPad v9.3.1 Software (California Corporation, USA), Luminex-100 software version 1.7 (Luminex, Austin, USA), and Attune Cytometric Software.

For manuscripts utilizing custom algorithms or software that are central to the research but not yet described in published literature, software must be made available to editors and reviewers. We strongly encourage code deposition in a community repository (e.g. GitHub). See the Nature Portfolio guidelines for submitting code & software for further information.

Data

Policy information about availability of data

All manuscripts must include a data availability statement. This statement should provide the following information, where applicable:

- Accession codes, unique identifiers, or web links for publicly available datasets
- A description of any restrictions on data availability
- For clinical datasets or third party data, please ensure that the statement adheres to our policy

All data are available upon reasonable request.

Human research participants

Policy information about studies involving human research participants and Sex and Gender in Research.

Reporting on sex and gender	Both sex are included and the sex is not considered in study design.
Population characteristics	For human mesenchymal stem cells: H96: 30-years-old female, H124: 19-years-old female, H126: 16-years-old male, and H127: 22-years-old male.<br>For human myoblasts: 2 Females and 4 Males
Recruitment	The donors for human mesenchymal stem cells were recruited from patients who were undertaken orthopaedics surgeries. For the myoblasts, the donors were recruited from patients who were undertaken uneventful hip or scoliosis surgery.
Ethics oversight	The Lariboisiere Hospital (CODECOH number: DC-2018-3366) and Myobank-AFM (Authorization N°AC-2013-1868, Ethics Committee number BB-0033-00012, norma NF S 96-900).

Note that full information on the approval of the study protocol must also be provided in the manuscript.

Field-specific reporting

Please select the one below that is the best fit for your research. If you are not sure, read the appropriate sections before making your selection.

☒ Life sciences ☐ Behavioural & social sciences ☐ Ecological, evolutionary & environmental sciences

For a reference copy of the document with all sections, see nature.com/documents/nr-reporting-summary-flat.pdf

Life sciences study design

All studies must disclose on these points even when the disclosure is negative.

Sample size	No sample size calculation was performed in this study. Sample sizes were determined according to previous publications. In vitro experiments were conducted at least in triplicate whereas in vivo experiments were conducted using at least 5 samples for each group of hydrogels tested.
Data exclusions	No data were excluded from the analyses.
Replication	The in vitro experiments were performed from at least three times independently. The animal experiments were performed in two batches.
Randomization	The nude mice were randomly allocated to control or experimental group.
Blinding	The operator was blinded to the sample allocation during animal model setup and outcome assessment as another investigator prepared the samples. In addition, the experimenters were blind during data collection and analyses.

Reporting for specific materials, systems and methods

We require information from authors about some types of materials, experimental systems and methods used in many studies. Here, indicate whether each material, system or method listed is relevant to your study. If you are not sure if a list item applies to your research, read the appropriate section before selecting a response.

Materials & experimental systems

n/a	Involved in the study	
☐	☒	Antibodies
☐	☒	Eukaryotic cell lines
☒	☐	Palaeontology and archaeology
☐	☒	Animals and other organisms
☒	☐	Clinical data
☒	☐	Dual use research of concern

Methods

n/a	Involved in the study	
☒	☐	ChIP-seq
☐	☒	Flow cytometry
☒	☐	MRI-based neuroimaging

Antibodies

Antibodies used	1. beta-2-microglobulin rabbit polyclonal antibody NCL-B2Mp (1/1000, Novocastra, Nanterre, France)

Antibodies used	Following antibodies are all from miltenyi Biotech, 10µl per 100µl sample.<br>2. CD45-PerCP, human monoclonal (clone: 5B1), 130-098-145<br>3. CD73-PE, human monoclonal (clone: AD2), 130-097-943<br>4. CD90-FITC, human monoclonal (clone: DG3), 130-097-930<br>5. CD105-Vioblue, human monoclonal (clone: 43A4E1), 130-099-667<br>6. Mouse IgG1-FITC monoclonal (clone: IS5-21F5), 130-098-847<br>7. Mouse IgG1-PE monoclonal (clone: IS5-21F5), 130-098-845<br>8. Mouse IgG1-Vioblue monoclonal (clone: IS5-21F5), 130-099-756<br>9. Mouse IgG2-PerCP monoclonal (clone: S43.10), 130-099-190
Validation	All antibodies for immunohistochemistry and flow cytometry are commercially available and were validated by the manufacturers as follows:<br>1. https://www.abcam.com/products/primary-antibodies/beta-2-microglobulin-antibody-ab175031.html<br>2. https://www.citeab.com/antibodies/2090191-130-098-145-cd45-percp-human-monoclonal<br>3. https://www.miltenyibiotec.com/GB-en/products/cd73-antibody-anti-human-ad2.html<br>4. https://www.citeab.com/antibodies/2090707-130-097-930-cd90-fitc-human-monoclonal<br>5. https://www.miltenyibiotec.com/GB-en/products/cd105-antibody-anti-human-43a4e1.html<br>6. https://www.miltenyibiotec.com/US-en/products/isotype-control-antibody-mouse-igg1-is5-21f5.html#conjugate=fitc:size=30-tests-in-60-ul<br>7. https://www.miltenyibiotec.com/US-en/products/isotype-control-antibody-mouse-igg1-is5-21f5.html#conjugate=pe:size=30-tests-in-60-ul<br>8. https://www.miltenyibiotec.com/US-en/products/isotype-control-antibody-mouse-igg1-is5-21f5.html#conjugate=vioblue:size=30-tests-in-60-ul<br>9. https://www.miltenyibiotec.com/US-en/products/isotype-control-antibody-mouse-igg2a-s43-10.html

Eukaryotic cell lines

Policy information about cell lines and Sex and Gender in Research

Cell line source(s)	Human mesenchymal stem cells were isolated from bone marrow tissue obtained as discarded tissue during routine surgery from four adult donors, including H96: 30-years-old female, H124: 19-years-old female, H126: 16-years-old male, and H127: 22-years-old male.<br>Human umbilical vein endothelial cells are commercial cell line that are purchased from LONZA.<br>Human myoblasts were obtained from anonymized human skeletal muscle samples from healthy donors (2 Females, 4 Males) as post-surgical res nullius in the course of uneventful hip or scoliosis surgery.
Authentication	Human mesenchymal stem cells were verified by surface CD markers and differentiation potentials. Other cells were not authenticated. The myoblasts were authenticated by by analysing aliquotes for CD56 expression, or fusion capacity as previously described (Vilquin et al., Gene Ther. 2005; 12: 1651-1662).
Mycoplasma contamination	Negative mycoplasma contamination.
Commonly misidentified lines<br>(See ICLAC register)	No commonly misidentified cell lines were used in this study.

Animals and other research organisms

Policy information about studies involving animals; ARRIVE guidelines recommended for reporting animal research, and Sex and Gender in Research

Laboratory animals	Ten-week-old female nude mice were obtained from Janvier Labs. All mice were maintained under SPF conditions in a controlled environment of 18-23 ℃, a 12/12 hours light/dark cycle, and 40-60% humidity.
Wild animals	No wild animals were used.
Reporting on sex	Only female mice were applied in this study, however, the sex is not considered in the study design.
Field-collected samples	No field-collected samples were used.
Ethics oversight	All procedures regarding housing, handling, surgery, and recovery of the animals after surgery were conducted in compliance with the new European Directive 2010/63/EU guidelines regarding the protection of animals used for scientific purposes. The surgery procedures had received approval from the Ethics Committee on Animal Research at the Lariboisiere/Villemin (Paris, France; number S85 / 2014-07-30; number S158 / 2018-09-07).

Note that full information on the approval of the study protocol must also be provided in the manuscript.

Flow Cytometry

Plots

Confirm that:

☒ The axis labels state the marker and fluorochrome used (e.g. CD4-FITC).

☒ The axis scales are clearly visible. Include numbers along axes only for bottom left plot of group (a 'group' is an analysis of identical markers).

☒ All plots are contour plots with outliers or pseudocolor plots.

☒ A numerical value for number of cells or percentage (with statistics) is provided.

Methodology

Sample preparation	The human mesenchymal stem cell-contaning hydrogels were incubated with both 1 µg/mL nucleic acid stain Hoechst 33342 (HE; Sigma-Aldrich) and 1 µg/mL propidium iodide (PI; Sigma-Aldrich) at 37°C for 20 minutes. Hydrogels were then digested, and hMSCs were detached from hydrogels using trypsin-EDTA for 20 min. Then PBS containing 2% bovine serum albumin (BSA, Sigma-Aldrich) was added to stop the chemical action of trypsin. After centrifugation (at 3,500xg for 5 min), the hMSCs were re-suspended in fresh PBS and analyzed using an Attune flow cytometer (Life Technologies, Saint Aubin, France).
Instrument	Attune flow cytometer.
Software	Attune Cytometric Software.
Cell population abundance	No sorting were performed in this study.
Gating strategy	Cells were defined based on FSC-A and SSC-A parameters and cell aggregates were excluded using FSC-H versus FSC-A signal. Subsequently, viable cells were further defined by the absence of staining with viability dye. In details, cells staining both HE positive and PI negative were identified as "viable cells", whereas those staining both HE positive and PI-positive were identified as "dead cells".

☐ Tick this box to confirm that a figure exemplifying the gating strategy is provided in the Supplementary Information.
